# Serum and Urine Biomarker Leucine-Rich Alpha-2 Glycoprotein 1 Differentiates Pediatric Acute Complicated and Uncomplicated Appendicitis

**DOI:** 10.3390/diagnostics11050860

**Published:** 2021-05-11

**Authors:** Mohit Kakar, Marisa Maija Berezovska, Renars Broks, Lasma Asare, Mathilde Delorme, Emile Crouzen, Astra Zviedre, Aigars Reinis, Arnis Engelis, Juta Kroica, Amulya Saxena, Aigars Petersons

**Affiliations:** 1 Department of Pediatric Surgery, Children’s Clinical University Hospital, LV-1004 Riga, Latvia; marisamaija@gmail.com (M.M.B.); astra.zviedre@rsu.lv (A.Z.); arnis.engelis@rsu.lv (A.E.); aigars.petersons@rsu.lv (A.P.); 2 Department of Pediatric Surgery, Riga Stradins University, LV-1007 Riga, Latvia; 3 Department of Biology and Microbiology, Riga Stradins University, LV-1007 Riga, Latvia; renars.broks@rsu.lv (R.B.); aigars.reinis@rsu.lv (A.R.); juta.kroica@rsu.lv (J.K.); 4 Statistical Unit, Riga Stradins University, LV-1007 Riga, Latvia; lasma.asare@rsu.lv; 5 Faculty of Medicine, Riga Stradins University, LV-1007 Riga, Latvia; mathildedelorme1@gmail.com (M.D.); emilecrouzen@gmail.com (E.C.); 6 Department of Pediatric Surgery, Chelsea Children’s Hospital, Chelsea and Westminster NHS Fdn Trust, Imperial College London, London SW10 9NH, UK; Amulyasaxena@gmail.com

**Keywords:** pediatric appendicitis, biomarkers, serum LRG1, urine LRG1

## Abstract

Purpose: This prospective, single-center cohort study analyzes the potential of inflammatory protein mediator leucine-rich alpha-2 glycoprotein 1 (LRG1) for the early and accurate diagnosis of acute appendicitis (AA), and differentiation of acute complicated (AcA) from uncomplicated appendicitis (AuA). Methods: Participants were divided into the AcA, AuA, and control groups, and their serum (s-LRG1) and urine LRG1 (u-LRG1) levels were assayed preoperatively on the second and fifth postoperative days. Results: 153 patients participated, 97 had AA. Preoperative u-LRG1 with a cut-off value of 0.18 μg/mL generated an area under the receiver operated characteristic (AUC) curve of 0.70 (95% CI 0.62–0.79) for AA versus control (*p* < 0.001), while the results for AcA versus AuA were not significant (AUC 0.60, 95% CI 0.49–0.71, *p* = 0.089). The s-LRG1 levels of AA versus the control with a cut-off value of 51.69 μg/mL generated an AUC of 0.94 (95% CI 0.91–0.99, *p* < 0.001). The cut-off value of s-LRG1 was 84.06 μg/mL for diagnosis of AcA from AuA, and therefore, significant (AUC 0.69, 95% CI 0.59–0.80, *p* = 0.001). Conclusions: LRG1 exhibited excellent diagnostic performance as an inexpensive, non-invasive, rapid, and accurate biomarker able to reflect the pathogenesis of AA. LRG1 has the potential to replace advanced imaging to diagnose clinically ambiguous AA cases.

## 1. Introduction

Acute appendicitis (AA) is the most common pediatric surgical emergency, which may result in abscess, peritonitis, sepsis, ileus or death due to delayed diagnosis and treatment [1,2,3]. AA diagnosis currently depends on typical clinical findings and anamnestic evidence despite the advancement of various diagnostic techniques. The recent interest and evidence of non-surgical treatment with antibiotic therapy leads to the recurrent issue of differentiating acute uncomplicated appendicitis (AuA) from acute complicated appendicitis (AcA) upon presentation in the emergency department. Swift confirmation of appendicitis is impeded by a variety of factors such as atypical presentation and multiple differential diagnoses, thus elevating the likelihood of complications. In Latvia, delayed diagnosis is caused by perpetuating diagnostic steps in more than 35% of the cases [4].

The Alvarado score is a frequently utilized tool for AA symptom gradation, yet it lacks specificity and sensitivity [5]. Although appendicitis is particularly common in children, the clinical history assessment is often marred by the lack of cooperation and fluency [6,7,8,9,10]. To promote accuracy, evaluation methods such as a computed tomography (CT) and diagnostic laparoscopy are applied, but nevertheless these are time inefficient, costly, and invasive (e.g., CT-radiation increasing the long-term cancer risk) [9]. Current diagnostics such as leukocytosis, increased serum C-reactive protein (CRP), and abdominal ultrasound (US) imaging have assisted in decreasing the frequency of diagnostic laparoscopies [10]. Despite these diagnostic tools, current negative appendectomy rates of 4–45% have stimulated the search for a non-invasive strategy to lower these error figures, which has led to a rise of novel biomarkers being introduced for both the assessment and detection of appendicitis [1,11].

Multiple studies have revealed the success of the efficient diagnostic scheme provided by inflammatory biomarkers as a non-invasive analysis, increasing accuracy and speed of diagnosis, and reducing healthcare costs dramatically [12]. As immunological pathways are better understood, more biomarkers have been proposed as potential diagnostic tools, however, none are in widespread use. In comparison to Interleukin-6 (IL-6) and CRP, no other biomarkers have been proven effective in diagnosing appendicitis. The prospect of a biomarker with an even higher accuracy rate than those currently investigated is galvanizing.

Leucine-rich Alpha-2 Glycoprotein (LRG1) is a novel biomarker and is hypothesized to not only have a particularly vital and rapid diagnostic precision ratio, but also can determine specificity in acute appendicitis development with drug-independent serum-values [7,13,14]. Although its complete mechanism of action is still unclear, LRG is thought to play a role in the activation and chemotaxis of neutrophils as they enter areas of inflammation [13,14]. LRG-1 is a 50 kD membrane-associated acute phase protein of the Leucine-rich Repeat (LRR) motif, consisting of 312 amino acids— 66 of which are leucine [14,15]. LRG-1 is produced and secreted by hepatocytes, neutrophils, macrophages, and intestinal epithelium, enabling it to not be strictly contingent on any of these cells, and is upregulated in acute phase responses of microbial infections at inflammatory sites [13,14,15]. Its normal serum level is hypothesized to be 21–50 μg/mL [15].

Numerous pro-inflammatory markers such as IL-6, IL-1*β*, IL-22, TNF-*α*, and lipopolysaccharides stimulate the transcription of LRG1, therefore, it is not contingent on a singular stimulating factor [15]. Another peculiar facet of LRG1 is the remarkably increased concentration at the local site of inflammation, possibly distinguishing infections based on marked LRG1 deposition [15]. The current diagnostic issues of AcA show the necessity to find new early diagnostic indicators for pediatric patients to reduce the incidence of complications. The primary study objective is to demonstrate that LRG1 could potentially differentiate AcA from AuA in a prospective cohort study with cases of pediatric acute appendicitis.

## 2. Materials and Methods

### 2.1. Study Design and Setting

A prospective, single-center, randomized controlled cohort study consisted of children admitted to the Emergency Department (ED) at the Children’s Clinical University Hospital (CCUH) in Riga with suspected appendicitis. This tertiary hospital is the only facility in Latvia specialized in treating children, and more than 6000 patients annually are admitted to the ED. The study meets the basic principles of the Helsinki Declaration and the requirements of the Patient’s Data Protection Law. The Ethics Committee’s approval was received by both the Children’s Clinical University Hospital and Riga Stradins University (reference number: SP-37/2018 and 21/27.04.2017, respectively) between January 2017 and 2020, during which the research was conducted.

### 2.2. Study Population

Participants of this study were children between the ages of 7 and 17 who presented to the ED with suspected appendicitis. Control group participants were patients without any suspected inflammatory process in the urinary, gastrointestinal or respiratory tracts; therefore, the included ED admitted pediatric patients of similar ages treated for various traumas (e.g., fractures, dislocations, contusions, muscle tears, testicular torsion, blunt abdominal trauma). The exclusion criteria for suspected appendicitis patients involved prior abdominal surgery, pregnancy, and chronic medical and malignant conditions that could potentially affect the urinary, gastrointestinal, or respiratory systems (e.g., inflammatory bowel disease, chronic pancreatitis, acute kidney injury, and immunosuppressed patients). The allotment of approximately 150 patients limited the study to not include patients with non-specific abdominal pain, therefore, the focus needed to be determined whether the biomarker levels differ in appendicitis patients and patients without any gastrointestinal, urinary or respiratory tract afflictions. Patient group size was divided to be in equal amounts in order to limit the assumption of variances similar to the Levene’ s test.

### 2.3. Study Protocol

Each patient’s medical history, physical examination, Alvarado score, and biochemical blood analysis were collected by the treating physician per the hospital’s protocol (Nr. REK-052/01). If a patient with suspected appendicitis received an Alvarado score of six or more, a surgical consultation was prescribed and, if necessary, additional radiological imaging was considered, such as abdominal US and/or CT. Once appendicitis was confirmed and all inclusion criteria were met, a consent form was given to the patient and his or her caregiver. This consent form contained the research objective, methodology of biological materials used, and the process of biological material collection. This same consent form was given to caregivers and patients of the control group.

All participants were screened for LRG1 levels from serum and urine specimens on the day of admission, and subsequently on the second and fifth postoperative day for AA participants. The current trend for treating pediatric appendicitis to predominately perform minimal invasive techniques for surgical treatment and therefore, laparoscopic appendectomy is more common. The patients were operated by certified pediatric surgeons of the CCUH with a co-author as an assistant or the operating surgeon. Intraoperatively, microbiological cultures were obtained from the patient’s peritoneal cavity. After appendectomy, additional microbiological cultures were taken from the appendix and then the appendix was sent for further histological examination. Patients were objectively classified as AuA or AcA by the absence or presence of bacterial growth in the peritoneal cavity. The primary outcome was to determine whether there is a biomarker level difference between appendicitis and non-appendicitis patients; secondarily, whether this biomarker differentiated between AuA and AcA.

### 2.4. Serum LRG1 Collection and Analysis

Commercially available Human Leucine-rich alpha-2-Glycoprotein 1 ELISA kit (Catalog No. NBP2-60577, Novus Biologicals, USA) were used to determine LRG1 levels, per the manufacturer’s instruction manual. The minimum amount of blood required per patient for LRG1 was 300 μL per analysis (baseline, second and fifth postoperative day). The serum was collected and stored at −80 °C after centrifugation. All of the samples were measured in three different wells with results appearing within approximately 2–4 h. This kit used the ELISA method to detect human LRG1 that employs a quantitative sandwich enzyme immunoassay technique. The patient samples were sandwiched by the immobilized antibody and pre-coated LRG1-specific polyclonal antibody wells, and subsequently recognized by the streptavidin-peroxidase conjugate. After the unbound materials were washed away, a peroxidase enzyme substrate was added from which the color intensity could be measured. The minimum detectable LRG1 serum level was 0.313 ng/mL. Obtained values in ng/mL were converted to μg/mL for further calculations.

### 2.5. Urine LRG-1 Collection and Analysis

The same kit and instruction manual procedure were employed for urine analyses. Midstream, clean-catch urine specimens of at least 200 μL were collected in a sterile cup on admission, and subsequently for AA participants on the second and fifth postoperative day. Most AA patients had their day 0 urine sample collected during operation through a urine catheter. Urine samples were centrifuged at 2000 rpm for 10 min at 4 °C and then these supernatants were stored at −80 °C before analysis. The following day, the samples were transported to the laboratory for processing. The same steps of ELISA, as performed on s-LRG1, were also executed on these samples.

### 2.6. Statistical Analysis

Microsoft Excel 2016 and IBM SPSS Statistics 26 were utilized for statistical analyses and the data was validated by a statistical analyst for accuracy. Results were expressed as median values and interquartile ranges (IQR). The comparison between groups was calculated using the Mann–Whitney U-Test for two groups and Kruskal–Wallis test for all three groups for quantitative variables, which do not follow normal distribution. The Fisher Exact Test and Pearson Chi-square test were applied on qualitative variables to determine associations between the groups. A receiver operated characteristic (ROC) curve was generated by plotting the false-positive fraction versus the true-positive fraction for every possible cut-off score, and area under the ROC curve (AUC) was calculated; therefore, this determined the clinical importance of the biomarkers, as well as their diagnostic value regarding appendicitis. A *p*-value of <0.05 was considered statistically significant.

## 3. Results

### 3.1. Clinical Characteristics of the Study Population

During the study, samples were collected from 153 patients eligible for this research; 97 were diagnosed with appendicitis and 56 had no suspected infectious or inflammatory pathology. Participant ages ranged from 7 to 17 years, with a median of 12 years; 58% of which identified as male and 42% female. Patients with AA underwent appendectomy, and intraoperative swabs of free peritoneal fluid were collected to detect potential bacterial growth in the peritoneal cavity to distinguish AcA 52 (53.6%) from AuA 45 (46.4%).

Patients were predominately operated laparoscopically (87.6%), and only four AuA and eight AcA patients were operated conventionally. Nine AuA (22.5%) and 31 AcA (77.5%) patients required the placement of a drainage tube; 83 AA patients received an abdominal ultrasound, but only 69 patients’ diagnoses were confirmed for acute appendicitis. Demographics and clinical characteristics of the patients are presented in Table 1.

### 3.2. Serum Leucine-Rich Alpha Glycoprotein-1 Levels

**Figure 1:**
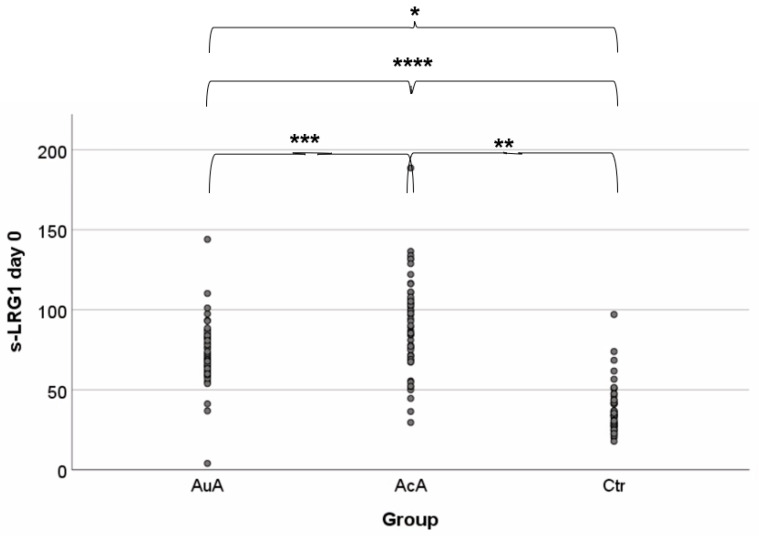
s-LRG1 of each patient group. s-LRG1 levels are increased in patients with AA (*n* = 97) compared to the control group (*n* = 56), **** *p* < 0.001. s-LRG1 could detect disease progress when analyzing AcA (*n* = 52) and AuA (*n* = 45), *** *p* = 0.001. A significant difference was recognized when the control group was compared to AcA and AuA separately; * *p* < 0.001 and ** *p* < 0.001, respectively.

Baseline preoperative s-LRG1 concentrations are presented in Table 2, along with values of the second and fifth postoperative days. s-LRG1 median value is more than twice as high in AuA compared to control group and almost three times as high in AcA. The median preoperative s-LRG1 values for AcA, AuA, and the control group were 88.12 μg/mL, 70.56 μg/mL, and 34.08 μg/mL respectively. Additional assessment of the dependency between s-LRG1 concentration and disease grade in AA patients is demonstrated in Figure 1, which reveals that appendiceal mucosal inflammation significantly correlates with an increased s-LRG1. There was a significant difference between control and AcA and/or AuA (*p* < 0.001, *p* < 0.001), as well as disease severity (AcA versus AuA) *p* = 0.001 when compared between AcA vs. AuA only.

s-LRG1 levels declined to 80.97 μg/mL and 66.73 μg/mL in AcA and AuA (*p* = 0.110) respectively on the fifth postoperative day, which were also significantly lower than levels at ED admission (*p* < 0.001) (Table 2). Thus, these results suggest s-LRG1, as a novel biomarker after appendectomy, correlates with patient recovery.

### 3.3. Urine Leucine-Rich Alpha-2 Glycoprotein-1 Levels

**Figure 2:**
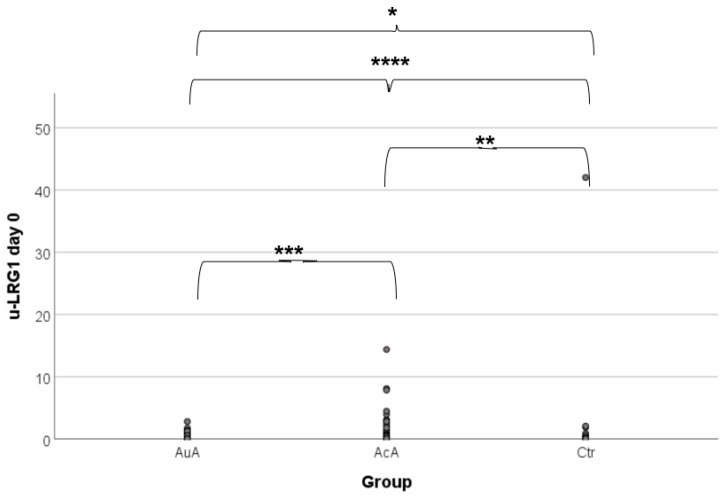
u-LRG1 of each patient group. u-LRG1 levels are increased in patients with AA (*n* = 97) compared to the control group (*n* = 56), **** *p* < 0.001. u-LRG1 was not able to detect disease progress when analyzing AcA (*n* = 52) and AuA (*n* = 45), *** *p* = 0.089. A significant difference was recognized when the control group was compared to AcA and AuA separately; * *p* < 0.001 and ** *p* = 0.005, respectively.

Baseline preoperative values and values on the second and fifth postoperative days of u-LRG1 are shown in Table 2. The median preoperative u-LRG1 levels for AcA, AuA, and control group were 0.35 μg/mL, 0.10 μg/mL, and 0.04 μg/mL respectively; therefore, the control group presented with the lowest baseline level. Further assessment of whether u-LRG1 levels were associated with disease activity in patients with AA is demonstrated in Figure 2. This reveals that appendiceal mucosal inflammation significantly correlates with increased u-LRG1 levels (*p* = 0.001). There was a significant difference between control versus AcA and AuA (*p* < 0.001, *p* = 0.005); however, disease severity (AcA vs. AuA) could not be differentiated (*p* = 0.089).

u-LRG1 levels on the fifth postoperative day declined to 0.10 μg/mL in AcA and 0.04 μg/mL AuA (*p =* 0.102). u-LRG1 levels were significantly higher at the time of admission to the ED than on the fifth postoperative day, (*p <* 0.001) (Table 2). u-LRG1 concentrations dropped by more than 50% (52/93 patients) after the diseased appendix was resected. Thus, these results suggest u-LRG1, as a novel biomarker, correlates with the improvement of patient recovery post-appendectomy.

### 3.4. Threshold Sensitivity and Specificity of Leucine-Rich Alpha-2 Glycoprotein-1

**Figure 3:**
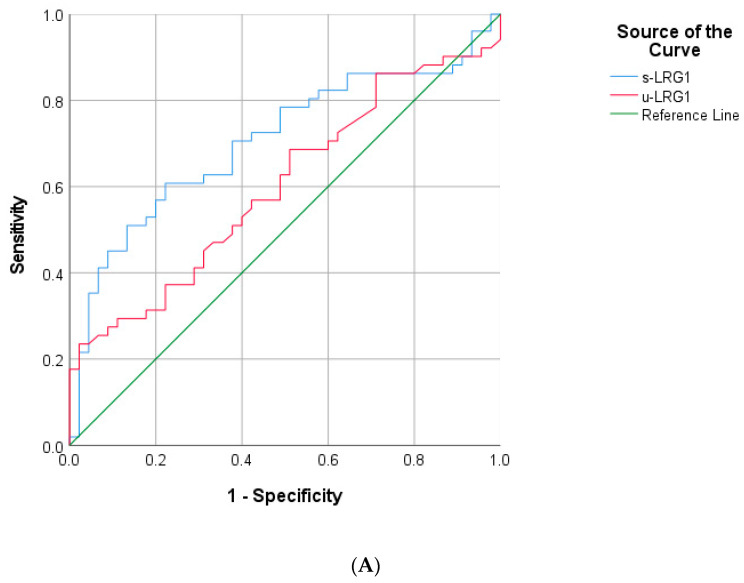

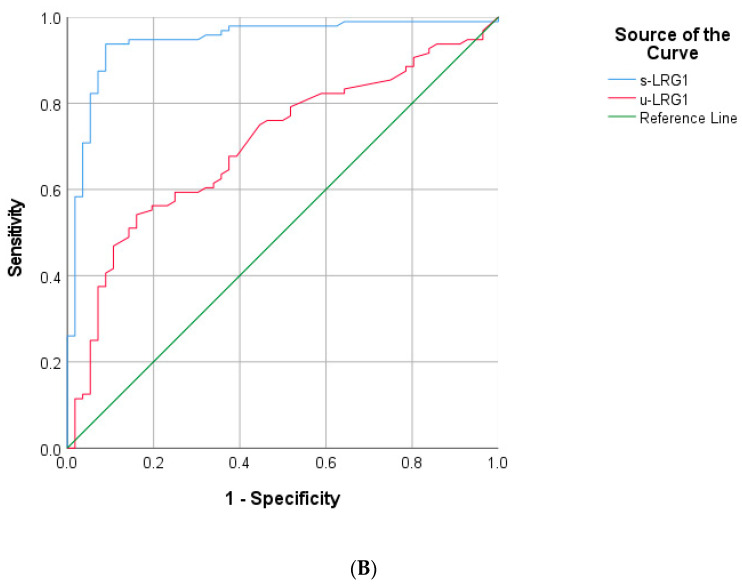
Analysis of s-LRG1 and u-LRG1 for sensitivity and specificity. The ROC curve for s-LRG1 analysis AcA vs. AuA children demonstrated AUC of 0.69 (95% CI 0.59–0.80) at cut-off value of 84.05 µg/mL and for u-LRG1 AUC of 0.60 (95% CI 0.49–0.71): (**A**) The ROC curve for s-LRG1 analysis AA vs. Ctr demonstrates AUC of 0.95 (95% CI 0.91–0.99) at cut-off value of 51.69 µg/mL and for u-LRG1 AUC of 0.70 (95% CI 0.62–0.79) at cut-off value of 0.175 µg/mL (**B**).

Given the potential role of LRG1 as a biomarker for clinical diagnosis of AA, additional investigation of its diagnostic accuracy in detecting the complicated course of disease was performed by analyzing sensitivity and specificity of serum and urine LRG1 through the application of ROC curves and AUC analysis. The s-LRG1 cut-off value for patients with AA was 51.69 μg/mL, and for AcA was 84.05 μg/mL. The ROC curves demonstrate an AUC of 0.95 (95% CI 0.91–0.99) and 0.69 (95% CI 0.59–0.80) respectively (Figure 3A,B). s-LRG1 in AA had 93.8% sensitivity and 91.1% specificity, while in AcA, it displays a lower sensitivity and specificity: 59.6% and 77.8%.

The u-LRG1 cut-off value for AA patients was 0.175 μg/mL, and for AcA was not significant. The ROC curves demonstrate an AUC of 0.70 (95% CI 0.62–0.79) and 0.60 (95% CI 0.49–0.71) respectively (Figure 3A,B). u-LRG1 for appendicitis had a sensitivity of 54.2% and specificity of 83.9%. The efficiency of u-LRG1 as a predictive marker of AcA versus AuA was found to be insignificant (*p* = 0.089) (Figure 3A). Contrarily, u-LRG1 proves to be an adequate predictive marker of AA (*p* < 0.001) (Figure 3B).

## 4. Discussion

Accurate, early diagnosis of acute appendicitis continues to be a major diagnostic challenge, and biomarkers potentially can confirm appendicitis and determine the severity. Current inflammatory markers of leukocytosis and CRP are too vague to accurately determine appendicitis with high specificity or sensitivity [5]. The authors of this study assessed LRG1 as a potential diagnostic tool because its marked expression is believed to represent an increased inflammatory process in patients with appendicitis. The primary objective was to evaluate the significant potential of LRG1 in distinguishing AcA from AuA as to commence adequate therapy. This study demonstrated that patients with appendicitis had significantly elevated s-LRG1 and u-LRG1 concentrations compared to the control group and had a significant difference of s-LRG1 concentrations in AcA and AuA, indicating that s-LRG1 correlates with the severity of appendicitis.

This study met many limitations as testing was performed at a single-center with a finite range of resources during inconsistent hours which limited patient participation. Most patients received either antibiotic and/or intravenous fluid therapy prior to surgery and sample collection, which may have affected biomarker concentrations. The usage of different Human Leucine-rich alpha-2-Glycoprotein 1 ELISA kits by other publications make the comparison with the results of this study arduous. The analysis of LRG1 samples was performed at a clinical research laboratory, not at the hospital’s laboratory, and therefore, concentrations could have been affected. The required duration to process urine and serum LRG1 in a clinical care setting has been reported to be two hours by Salö et al. with the new generation ‘Ultrafast ELISA Assay’ [16]. Patient quota limited the multiple types of patients that could be included; however, this study provided a basis for future broader studies, which are already underway. Patients under seven were excluded—as that age range tends to have a different pathophysiology for appendicitis as there is a strong link to bouts of viral infections such as gastroenteritis—and pre-hospitalization anamnestic information can be quite limited. The authors already have started another research study which includes these young patients.

A diagnosis of appendicitis needs to be ruled out from non-specific abdominal pain, and quite frequently, laboratory analyses and a surgical consult are the tools. Patients with non-surgical abdominal pain were not included in this study due to the patient quota. It was important to determine whether there was any basis for expanding this type of study regarding LRG1 diagnostic abilities in acute appendicitis. Multiple recent publications demonstrate the correlation between an enriched s-LRG1 concentration and the confirmed diagnosis of AA in children, which also is supported by this study’s findings [7,12,13,14,17,18,19]. A vast amount of clinical reports observes increased s-LRG1 levels in the development of a variety of disorders such as inflammatory disorders (intestinal, renal, and respiratory systems), oncological pathology (e.g., colorectal cancer, hepatocellular carcinoma, pancreatic cancer, ovarian cancer, lung cancer) and other chronic conditions (e.g., ulcerative colitis (UC), hydrocephalus, heart failure) [15,20,21,22,23,24,25,26,27,28,29]. The presence of LRG1 in diseased appendices and serum can be explained by neutrophils secreting LRG1 in response to bacteria [12,14,19,25,30]. Its role in inhibiting cell apoptosis by binding to Cytochrome C stimulates lymphocyte survival in the appendix and protects appendiceal tissue from susceptibility to toxicity [31]. In addition, LRG1 binds to accessory receptors of Transforming Growth Factor-*β* regulating a signaling pathway that stimulates angiogenesis, which may enhance tissue inflammation [25,30]. These studies also did not include patients with non-specific abdominal pain and therefore, further investigation is warranted.

Yap et al. revealed the efficient combination of s-LRG1 analysis with Alvarado score assessment had improved diagnostic accuracy of AA, creating the possibility of diagnosing AA at the ED without radiological confirmation [19]. The data show that s-LRG1 has markedly high specificity and sensitivity for the confirmation of suspected AA (Figure 3B), which leads to agreement with the suggested strategy of Yap et al. to replace IL-6 with s-LRG1 in the Alvarado score with a proposed lower limit of 51.69 μg/mL as a standard for suspect AA. Anupam et al. produced a cut-off value of 40.15 μg/mL [30].

Time is a vital factor in the progression of AA. Subacute appendicitis cannot be detected by CRP, IL-6 or leukocytes. Neutrophils act as first responders to infection, which explains the rapid detection of AA by LRG1, as it is secreted by neutrophils. Additionally, LRG1 has a longer half-life than CRP, thus the time range in AA is in favor of LRG1 [15]. CRP production is primarily dependent on liver stimulation by IL-6, whereas LRG1 is secreted by hepatocytes, intestinal epithelium, neutrophils, and macrophages if induced by IL-22, IL-1*β*, TNF-α and LPS apart from IL-6 [13,14,15,30]. Markedly high s-LRG1 is immediately associated with high vulnerability to a complicated appendicitis and hence is indicated for emergency appendectomy. Elevation of s-LRG1, to an ambiguous extent with low potential of AcA, grants valid grounds for antibiotic therapy. It was demonstrated in this study that s-LRG1 levels lowered in response to treatment in patients’ post-appendectomy and receiving antibiotic therapy. To evaluate positive treatment response, s-LRG1 can be re-evaluated on second and fifth day of antibiotic therapy to exclude the necessity of invasive treatment. This proposal may aid future approach to conservative AA treatment strategy.

u-LRG1 levels of pediatric appendicitis patients were significantly elevated in comparison to the control group in this study (0.10–0.35 μg/mL and 0.04 μg/mL respectively, *p* < 0.001). This confirms previously published statements on LRG1 as an accurate diagnostic method, superior to current urinary inflammatory markers [13,14,16,17,32]. From Figure 3, the authors deduct that starting from a u-LRG1 value of 0.175 μg/mL could indicate AA. Anupam et al. generated a cut-off value of 0.042 μg/mL using an alternative ELISA [30].

The renal threshold and selective filtering of LRG1 are unknown, but its marked elevation in urine proposes its local release by inflammatory sites (e.g., mesoappendix) and/or neutrophils [12,14,33]. Lee et al. explained LRG1 expression in urine by analyzing renal tubular injury caused by proteinuria, which induces an NLRP3 activation and maturation of IL-1*β*, and therefore, results in a positive stimulation of LRG1 in tubular epithelial cells in various renal disorders [22]. It is necessary to investigate more on the physiology of LRG1 in the kidney as it is increased in AA cases, which typically do not have renal impairment. Rodriguez-Suarez et al. found that, beside the merit of LRG1 to AA diagnosis, their results of increased u-LRG1 levels corresponded to the histological severity of AA [32]. When they applied Selected Reaction Monitoring (SRM), their results demonstrated that u-LRG1 is elevated 100-fold in patients with acute appendicitis compared to those without [32]. This study’s results with ELISA merely showed a mild significant difference between AA and control, and thus, when comparing AcA and AuA, there was no sufficient difference (0.42 and 0.33 μg/mL respectively, *p* = 1.000).

The physiological characteristics of LRG1 include the ability to distinguish AA severity, its rapid response to inflammation, resolute accuracy, enduring half-life, and independence from stimulatory and secretory mechanisms. Therefore, LRG1 qualifies as a more adequate and efficient biomarker compared to those currently applied. It should be emphasized that the results of the study in Latvia could improve the quality of medical care in relatively low socio-economic situated countries. Thus, urinalysis of LRG1 as an inexpensive, non-invasive diagnostic tool that could substitute imaging techniques, altering present diagnostic guidelines. However, prospective studies in large populations should consider other inflammatory markers so as to increase diagnostic efficacy of severity of appendicitis. The accuracy of u-LRG1 and s-LRG1 in diagnosing AA should be more elaborately investigated, focusing on its pathophysiology (e.g., in the renal and intestinal tract). LRG1 deserves a wider research scope in various institutions to continue its verification as a valuable diagnostic tool in AA; the approach of which must involve the exclusive use of one type of Human Leucine-rich alpha-2-Glycoprotein 1 ELISA kit. The response of LRG1 to antibiotic treatment promises interesting possibilities of altering therapy evaluation. Additional investigations remain to be completed regarding the response of LRG1 to diverse types of bacteria and different classes of antibiotic and intravenous fluid therapy. The fact that s-LRG1 is significantly increased in AcA when compared to AuA, enables speculation of its spread to the peritoneal fluid, which, thus far, is insufficiently examined. Cut-off values of s-LRG1 or u-LRG1 for the determination of AA or AcA need to be investigated further so a standard of diagnostic LRG1 values may be confirmed. Furthermore, the potential of LRG1 mRNA as an accurate marker for AA should be investigated.

## 5. Conclusions

The results suggest that along with clinical suspicion of AA, LRG1 is an accurate marker in diagnosis confirmation. The severity of AA with respect to AcA and AuA can be distinguished by the s-LRG1 concentration, and therefore, the evaluation of s-LRG1 could prove useful to initiate adequate therapy and analysis of disease status. u-LRG1 and s-LRG1 exhibited excellent diagnostic performances as inexpensive, non-invasive, rapid, and accurate biomarkers that reflect the pathogenesis of AA. The results of this study could improve the quality of medical care in relatively low socio-economic countries.

## Figures and Tables

**Table 1:** Demographics and clinical characteristics of cohort population.

	AuA	AcA	Ctr	Total	*p*-Value
	*n* = 45	*n* = 52	*n* = 56	*n* = 153	
Gender, *n* (%)					
Boy	23 (14.4)	27 (18.3)	39 (25.5)	89 (58.2)	0.081 *
Girl	23 (15.0)	24 (15.7)	17 (11.1)	64 (41.8)	
Age, Mdn (IQR)	13.0 (10.0–15.0)	12.0 (9.0–14.0)	13.5 (10.3–15.0)	-	0.101 ***
Type of surgery, *n* (%)				85 (87.6)	
Laparoscopy	41 (91.1)	44 (84.6)	-		0.333 *
Laparotomy	4 (8.9)	8 (15.4)	-	12 (12.4)	
Ultrasound, *n* (%)	30 (43.5)	39 (56.5)	-	69 (0.83)	0.349 *
Drainage tube, *n* (%)				40	
Yes	9 (20.9)	31 (60.8)	-		<0.001 *
No	34 (79.1)	20 (39.2)	-	54	
Length of hospital stay, days (IQR)	5 (4–6)	6 (4–9)	-	-	0.002 **

AcA = Acute complicated appendicitis, AuA = Acute uncomplicated appendicitis, Ctr = control group. Median values are presented with IQR (25%; 75%). *—Pearson Chi-square test, **—Mann–Whitney U-test, ***—Kruskal–Wallis test.

**Table 2:** Pre- and post-operative LRG1 levels per appendicitis status.

	AcA, μg/mL (IQR)	AuA, μg/mL (IQR)	Control, μg/mL (IQR)	*p*-Value
**DAY 0**				
Serum	70.56 (62.64–83.43)	88.12 (71.12–106.13)	34.08 (27.50–42.37)	<0.001
Urine	0.10 (0.03–0.73)	0.35 (0.05–1.38)	0.04 (0.02–0.10)	<0.001
**DAY 2**				
Serum	74.99 (61.00–96.03)	87.90 (70.32–104.10)		0.048
Urine	0.08 (0.03–0.28)	0.21 (0.06–0.98)		0.017
**DAY 5**				
Serum	66.73 (56.98–85.28)	80.97 (62.14–99.03)		0.110
Urine	0.04 (0.02–0.27)	0.10 (0.03–0.25)		0.102

AcA = Acute complicated appendicitis, AuA = Acute uncomplicated appendicitis, LRG1 = Leucine-rich alpha glycoprotein-1. Median values are presented with IQR (25%; 75%).

## Abbreviations

AA	Appendicitis
AcA	Acute complicated appendicitis
AuA	Acute uncomplicated appendicitis
ELISA	Enzyme-linked immunosorbent assay
IL-6	Interleukin 6
s-LRG1	Serum Leucine-rich alpha-2 glycoprotein 1
TNF-*α*	Tumor necrosis factor-alpha
u-LRG1	Urine Leucine-rich alpha-2 glycoprotein 1
